# The evaluation of cytotoxicity and cytokine IL-6 production of root canal sealers with and without the incorporation of simvastatin: an invitro study

**DOI:** 10.1186/s12903-022-02039-y

**Published:** 2022-01-11

**Authors:** Apoorva Sharma, Kavitha Sanjeev, Vinola M. J. Selvanathan, Mahalaxmi Sekar, Nikhil Harikrishnan

**Affiliations:** Department of Conservative Dentistry and Endodontics, SRM Dental College, Ramapuram, Chennai, Tamil Nadu 600089 India

**Keywords:** Cytokine, Cytotoxicity, IL-6 receptor, Inflammation, Root canal sealers, Simvastatin

## Abstract

**Background:**

Freshly mixed root canal sealers when proximate the periapical tissues, trigger varying degrees of cytotoxicity/inflammatory reactions. Simvastatin, a class of the drug statin, is a widely used cholesterol-lowering agent with additional anti-inflammatory activities. This study assessed the effects of simvastatin on cytotoxicity and the release of IL-6 (Interleukin-6) production when incorporated in zinc oxide eugenol and methacrylate resin-based sealers.

**Methods:**

Experimental groups consisted of conventional zinc oxide eugenol and methacrylate based-EndoREZ sealers (ZE & ER respectively) and 0.5 mg/mL simvastatin incorporated sealers (ZES & ERS). L929 mouse fibroblast cells were exposed to freshly mixed experimental sealers and evaluated for cytotoxicity (MTT assay) and inflammation levels (inflammatory marker IL-6 for ELISA) at various time intervals (0h, 24h and 7th day). The values were compared to the cell control (CC; L929 cells alone) and solvent control (SC; L929 cells + DMSO) groups. All the experiments were conducted in triplicates and subjected to statistical analysis using IBM SPSS Statistics software. Non parametric tests were conducted using Kruskal-Wallis and Friedman tests for inter-group and intra-group comparisons respectively. Pairwise comparison was conducted by post hoc Dunn test followed by Bonferroni correction. *P* values < 0.05 were considered statistically significant.

**Results:**

All the experimental groups (ZE, ER, ZES, ERS) exhibited varying degree of cytotoxicity and IL-6 expression compared to the control groups CC and SC. The cell viability for ZE and ER decreased on day 7 as compared to 24 h. ZES and ERS had higher viable cells (75.93% & 79.90%) compared to ZE and ER (54.39% & 57.84%) at all time periods. Increased expression of IL-6 was observed in ZE & ER (25.49 pg/mL & 23.14 pg/mL) when compared to simvastatin incorporated ZE & ER (ZES-12.70 pg/mL & ERS-14.68 pg/mL) at all time periods. Highest level of cytotoxicity and inflammation was observed in ZE compared to all the other groups on day 7.

**Conclusions:**

Addition of 0.5 mg/mL of simvastatin to the sealers (ZES and ERS) decreased the cytotoxicity in the freshly mixed state and reduces their inflammatory effect.

## Background

Periapical tissue reactions following root canal treatment/or obturation are influenced by numerous factors, including pre-existing disease, removal of pulp tissue, cleaning and shaping of the root canal system, obturation technique and the chemical nature of the sealer [1]. Several studies have emphasized that root canal filling materials should be confined within the intra- radicular space [2, 3]. However, there are circumstances when controlled application is not possible and it may inadvertently extrude into the periradicular area thorough lateral and accessary canals, and apical foramen [4–6]. Evidences claim that, in absence of infections, though the apical extent of the root canal filling materials does not have direct correlation to the treatment outcomes, an inflammatory response with increased postoperative discomfort of varying intensity generally develop in areas where the sealers proximate the apical and periradicular tissues [4–6]. Amongst the various commercially available sealers, zinc oxide eugenol (ZOE) and methacrylate-based sealers (EndoREZ) have evidently shown the highest cytotoxicity, inducing periapical inflammation [7].

During periapical inflammation, along with inflammatory cells namely PMNs (polymorphonuclear leukocyte), lymphocytes and macrophages, there is also an increased release of proinflammatory cytokines, namely interleukins (ILs) [8, 9]. Following infections and tissue injuries, interleukin 6 (IL-6), is produced immediately and transiently, contributing to host defence through the stimulation of acute phase responses, haematopoiesis and immune reactions [10]. Azuma et al., reported detection of significantly higher levels of IL-6 in in vitro model, and in human inflamed pulp tissue and periapical lesions of endodontic origin. Therefore, IL-6 has been speculated to be a critical factor in cytokine cascade determining inflammation [11]. Successful endodontic treatment thus necessitates preventing and controlling this inflammation providing a favourable environment for periapical repair and healing.

Statins, 3-hydroxy-3-methyl-glutaryl-coenzyme A (HMG CoA) reductase inhibitors commonly used as an anticholestral drug, are reported to exhibit immunomodulatory, anti-inflammatory and anti-oxidative effects [12, 13]. Simvastatin a class of drug statin has proven to reduce the cytokine-mediated IL-6 release in mononuclear cells [14]. Sakoda et al*.*, showed the anti-inflammatory effect of simvastatin on human oral epithelial cells and found decreased IL-6 and IL-8 production [15]. Varying methodological approaches including observational, in vitro, animal, in vivo, randomized clinical studies and meta-analysis have concluded simvastatin’s effectiveness in chronic periodontitis when used locally and systemically, in non-surgical, surgical periodontal therapy and as oral administration, as an antimicrobial agents against oral microorganisms [16, 17]. Various animal model-based studies and clinical trials examined the effect of statins on the pathogenesis of periapical lesions and were conclusive about the role of statins in bone formation for e.g. in osseointegration of implants, as a local application in extraction sites to prevent alveolar bone resorption [16, 18–20]. Collectively, literature evidence of various clinical trials states that, rather than systemic administration, local application of statins have significantly enhanced beneficial effects on dental and oral health [16, 17]. Apart from its anti-inflammatory effect, recent widespread evidence proposes that, statins increase the gene expression of BMP-2 (bone morphogenic protein-2) and inhibit MMPs (matrix metalloproteins), thereby stimulating differentiation of osteoblastic bone marrow stem cells, enhancing wound healing [16, 21, 22]. Thus, the rationale aiding the use of statin through local administration accounts for its enhanced bioavailability.

In this context, whether the addition of simvastatin to the currently used root canal sealer is capable of reducing sealer induced cytotoxicity and inflammation needs to be evaluated. Hence the aim of this in vitro study was to comparatively evaluate the effects of the addition of simvastatin on the cytotoxicity and anti-inflammatory effect of ZOE and methacrylate sealer. The null hypothesis is that the addition of simvastatin will not reduce the cytotoxicity and inflammation caused by ZOE and methacrylate resin-based sealers.

## Methods

The research protocol was presented to Institutional Review Board (IRB) and approval was obtained SRMDC/IRB/2018/MDS/No. 301.

### Preparation of simvastatin

Dimethyl sulfoxide (DMSO), phosphate-buffered saline **(**PBS) and simvastatin powder were procured from Sigma-Aldrich St.Louis, MO, USA. 1:1 ratio of DMSO: PBS solution was prepared and set at pH 7.2. Simvastatin powder (0.5 mg) (Lot no: 0000048519,0000040533) was weighed using a digital weighing scale (BSA 224S CW, Sartorius, Gottingen, Germany.). The weighed simvastatin powder was incorporated into the prepared solution to obtain 0.5 mg/mL solution of simvastatin [6, 23, 24].

### Preparation of test samples

Equal amounts of base and catalyst of ZOE sealer (Tubli-seal, Kerr, Romulus, MI, USA, Lot no: 6807867) were dispensed on the mixing pad and mixed by spatulating the pastes for one minute to obtain 1 mL of the sealer (Group-ZE- Zinc oxide Eugenol). 1 mL of EndoREZ sealer (Lot no: BH 8 BC; Ultra dent Products, South Jordan, UT, USA.) was dispensed on the pad from the dual-barrel syringe through the mixing tip (Group-ER- EndoREZ) [6, 25]. The composition of the experimental sealers is mentioned in Table 1.

**Table 1:** Experimental sealers, their composition and manipulation

S. no	Sealers	Manufacturer’s company name	Lot no	Composition	Manufacturer’s instructions
1	Zinc Oxide Eugenol sealer (Tubliseal EWT)	Kerr, USA	6,807,867	Base: Zinc oxide Barium sulfate Lecithin Corn starch Mineral oil Catalyst: Polypale resin Eugenol Thymol Accelerator: 4-Allyl-2-methoxyphenol, Dimeric acid resin	Mix equal volume units (1:1) of base and catalyst
2	Methyl methacrylate sealer (EndoRez)	Ultradent Products, South Jordan, UT	BH 8 BC	Zinc oxide, Barium sulphate, Resins 30% UDMA resin, Pigments	Twist the dual barrel syringe counter clockwise to dispense the material

Groups ZES & ERS (simvastatin incorporated ZOE and Endo REZ sealers respectively) were prepared by incorporating 0.5 mg/mL simvastatin to ZOE and EndoREZ sealers respectively and spatulated 1 mL of Dulbecco Modified Eagle Medium (DMEM) (Gibco, Thermo Fisher Scientific, Waltham, MA, USA) with each of the sealer followed by placement in a cyclomixer (CM101 Plus, Remi, Mumbai, India) to obtain a uniform mix; 0.5 mL of this solution was taken and again mixed with 0.5 mL DMEM to obtain 1 mL of the sealer solution to be subjected to various evaluations [6].

Cytotoxicity and IL-6 assessment were carried out using MTT (3-(4,5-dimethylthiazol-2-yl)-2,5-diphenyltetrazoliumbromide) and ELISA (enzyme-linked immunoassay) assays respectively at various time intervals (0 h, 24 h and 7th day) for all the test samples.

### Cell culture

L929 mouse fibroblast cells (Lot no: ACC85011425; NCCS, Pune, India) were cultured using 25-cm^2^ culture flasks containing 2 mmol/L L-glutamine 10% fetal bovine serum, 100 µg/mL streptomycin, and 100 U/mL penicillin (all from Gibco, Thermo Fisher Scientific, Waltham, MA, USA). Cultures were kept in an incubator (BBD 6220; Thermo Fisher Scientific, Waltham, MA, USA) at 37 °C under ambient pressure and 5% CO_2_ atmosphere. Cells were used from the 3rd passage till the 20th passage [26]. Confluent cell monolayers were trypsinized, and the cells that were harvested, were used for cytotoxicity experiments.

### Cytotoxicity assessment

Assessment of the toxic effects of the tested materials on human periodontal ligament fibroblasts was performed using 3-(4,5-dimethylthiazol-2-yl)-2,5-diphenyltetrazoliumbromide (MTT assay), Live/ Dead staining using Calcein AM and Ethidium homodimer-1(EHD) and Flow cytometry following double staining with Calcein AM/ Propidium Iodide (PI).

#### MTT assay

This method enables determining cell viability and proliferation based on the mitochondrial activity of succinate dehydrogenase. Ninety six well plates (Costar, Corning, NY, USA) were taken for seeding exponentially growing L929 mouse fibroblast cells at a concentration of 1 × 10^4^ cells/well. After 24 h, the culture medium was cleared out and the cells were incubated in freshly prepared experimental sealers (0.5µL of the experimental solution added to the cells using a micropipette) for 24 h at 37 °C in an atmosphere of 5% CO_2_. For the experimental groups, cells were incubated with 0.2 mg sodium lauryl sulphate (Life Technologies, Mumbai, India) in DMEM medium, while for the cell control group (Group CC- L929 cells alone) and solvent control group (Group SC- L929 cells + DMSO), cells were incubated in culture medium alone and with DMSO and culture medium respectively. 50 µL of 0.5% 3-(4,5-dimethylthiazol-2-yl)-2,5-diphenyltetrazoliumbromide (Sigma- Aldrich, St. Louis, MO, USA) was added to each well and the plates were then incubated for approximately 2 h, at 37 °C in a humidified atmosphere of 5% CO_2_ in air. The conversion of the yellow 3-(4,5-dimethylthiazol-2-yl)-2,5-diphenyltetrazoliumbromide to the purple formazan by the cellular NAD(P) reflux was measured. MTT dye was removed after incubation, and 100 µL isopropanol (Life Technologies, Mumbai, India) was added to dissolve the formazan crystals. Plates were gently shaken at room temperature to ensure formazan solubilization, and transferred to the spectrophotometer (Multiskan Sky Microplate Spectrophotometer; Thermo Fisher Scientific, Waltham, MA, USA). The viability of the cultured cells was determined at a wavelength of 570 nm. All the experiments were done in triplicates [6].

The percentage of cell viability was then calculated as:

$${\text{Percentage of cell viability}} = \frac{{{\text{Absorbance of treated cells at 57}}0{\text{ nm}}}}{{{\text{Absorbance of control cells at 57}}0{\text{ nm}}}} \times 100\%$$

#### Live and dead cell assay

The advantage of the live/dead staining procedure applied, is that the respective red and green fluorescence of EHD and Calcein are easily discernable by florescence microscopy. L929 fibroblast cells were seeded in 6 well plates at the density of 1 × 10^6^ cells/well). After being cultured for 24 h, live/dead viability assay kit (Invitrogen Life Technologies, Carlsbad, CA). Cells were incubated with dye for 30 min and were washed with PBS. Live cells were stained green with 2 mmol/L Calcein AM (Invitrogen Life Technologies, Carlsbad, CA), and dead cells were marked red with 4 mmol/L ethidium homodimer-1. They were observed under inverted phase contrast fluorescence microscopy (20 × magnification). Viable cells exhibited green fluorescence while the dead cells appeared red. The percentage of the live cells were calculated using Image J software.

#### Flow cytometry analysis

The Live/Dead assay was determined by using Calcein-AM /Propidium iodide Double stain kit (G-Biosciences, USA). After incubation with control and experimental group of different composition of sealers, the cells (5 × 105) were harvested and suspended in 1 × phosphate buffered saline (PBS). Then the cells were incubated with Calcein-AM (50 µM)/propidium iodide (PI) (10 µg/ml) diluted in culture medium for 30 min in the dark. Cells were then washed with ice-cold 1 × PBS and immediately resuspended in sheath fluid in BD-FACS analysis tube for experiment. The FACS was performed using Becton–Dickinson canto II (BD Biosciences, USA) for analysis. The data were analyzed by FlowJo (version 7.6.1).

### Cytokine detection

The samples of groups were prepared according to the previous protocol. The collected culture suspensions were preserved in microtubes at − 20 °C. IL-6 kit (DY506, DuoSet; R & D systems, MN, USA) containing 96 well plates were used for determining the fibroblast cytokine level. Anti-IL-6-monoclonal antibody was added to each well of the ELISA plate. The samples of IL-6 were then conjugated with biotin and added to wells and were maintained for 2 h at room temperature. The samples were thoroughly rinsed with distilled water to eliminate any unbound compounds. Streptavidin HRP (Thermo Fisher Scientific, Waltham, MA, USA) was added to be bonded with the conjugated biotin-interleukin. After 1 h at room temperature, they were rinsed again with distilled water and the samples were assessed at 450 nm using spectrophotometer, IL-6 was assessed at 0 h, 24 h and 7th day. All the experiments were done in triplicates [27].

### Statistical analysis

At least three independent experiments were performed for each parameter and the mean value was used for statistical analysis. The data was analysed using IBM SPSS Statistics software (IBM Corp., Armonk, NY, USA). The results were presented as mean ± standard errors (SE). Parametric and Non parametric tests were conducted using students T test, Kruskal–Wallis and Friedman tests for inter-group and intra- group comparisons respectively. Pairwise comparison was conducted by post hoc Dunn test followed by Bonferroni correction. *P* values < 0.05 were considered statistically significant.

## Results

**Fig. 1:**
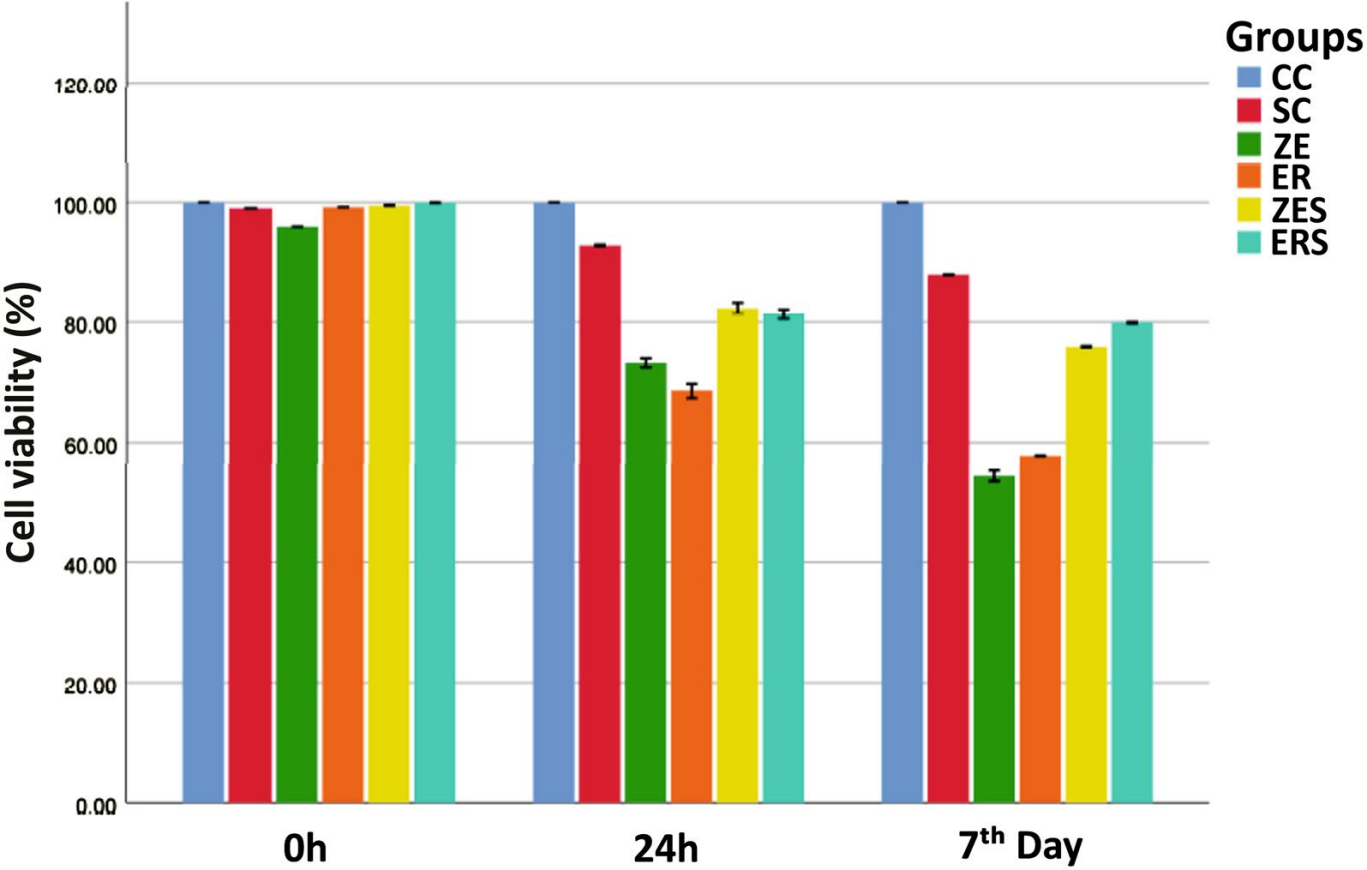
Graphical representation of percentage of cell viability of all the groups at various time intervals (Mean ± SD). Footnotes: h-hours CC-Cell control; SC-Solvent control; ZE-Zinc Oxide Eugenol sealer; ER-EndoREZsealer; ZES & ERS- Simvastatin incorporated Zinc oxide eugenol and EndoREZ respectively

**Fig. 2:**
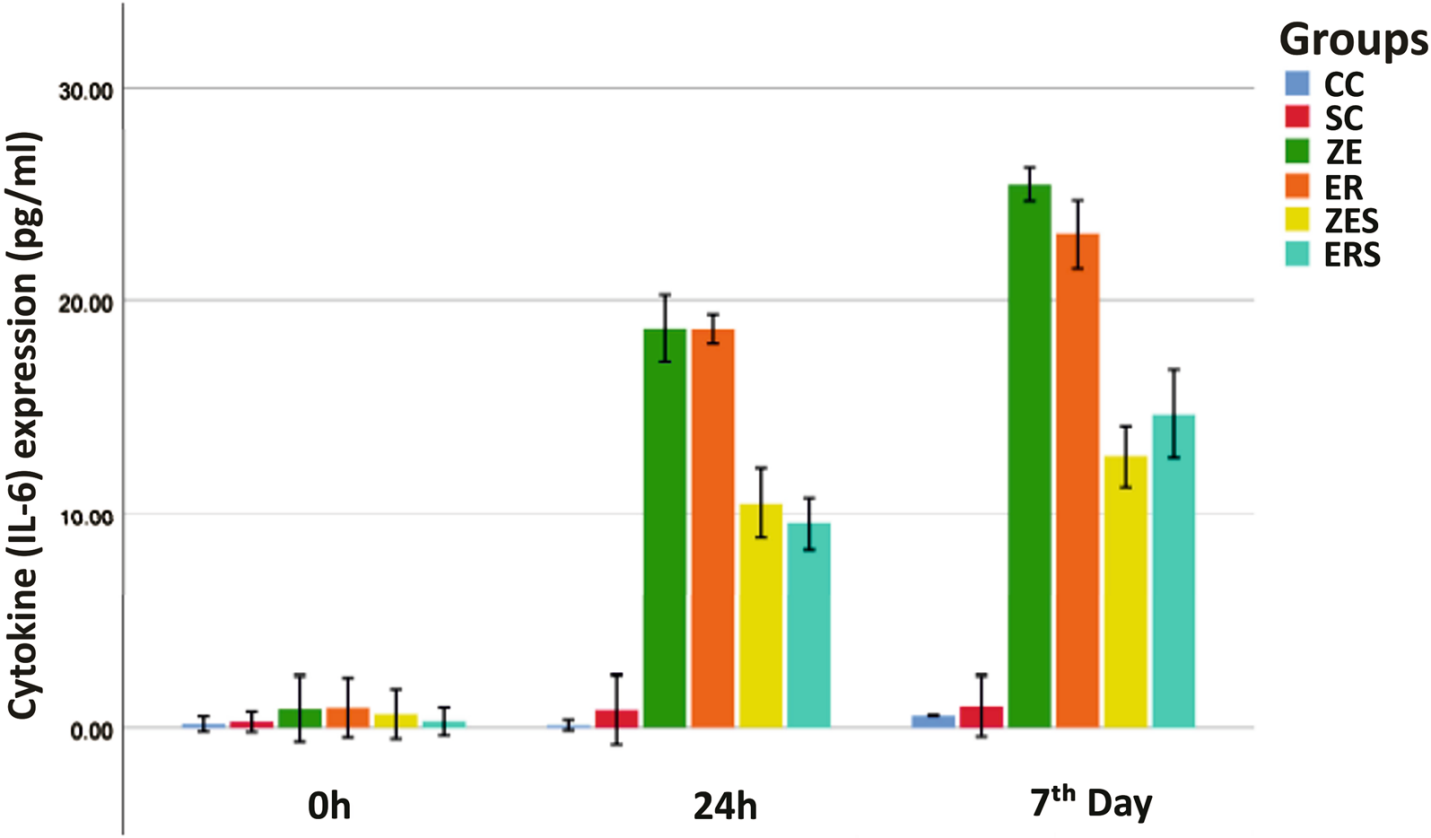
Graphical representation of cytokine (IL-6) expression (pg/mL) of all the groups at various time intervals (Mean ± SD). Footnotes: IL-6-Interleukin-6; pg/mL—picogram/millilitre h-hours; CC-Cell control; SC-Solvent control; ZE-Zinc Oxide Eugenol sealer; ER-EndoREZ sealer; ZES & ERS- Simvastatin incorporated Zinc oxide eugenol and EndoREZ respectively

**Fig. 3:**
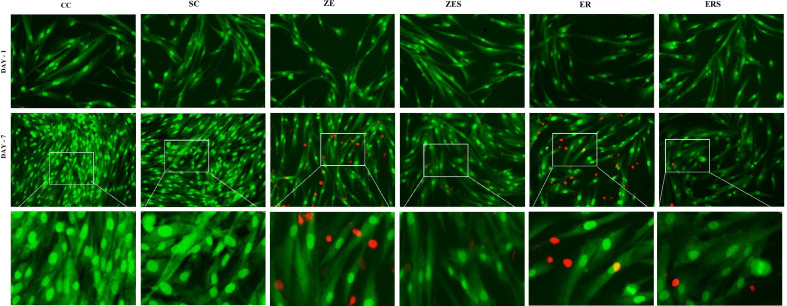
Live/dead assay: Representative live/dead cell images of experimental sealers with and without simvastatin on L929 cells at 24 h and day 7 of culture, observed under phase contrast fluorescence microscopy at 20 ×. The bottom row shows the enlarged images of 7th day. Live cells stained *green*, and dead cells shown in *red*. In all the groups, the live cells were abundant and few dead cells were noted in ZE,ER and ERS

**Fig. 4:**
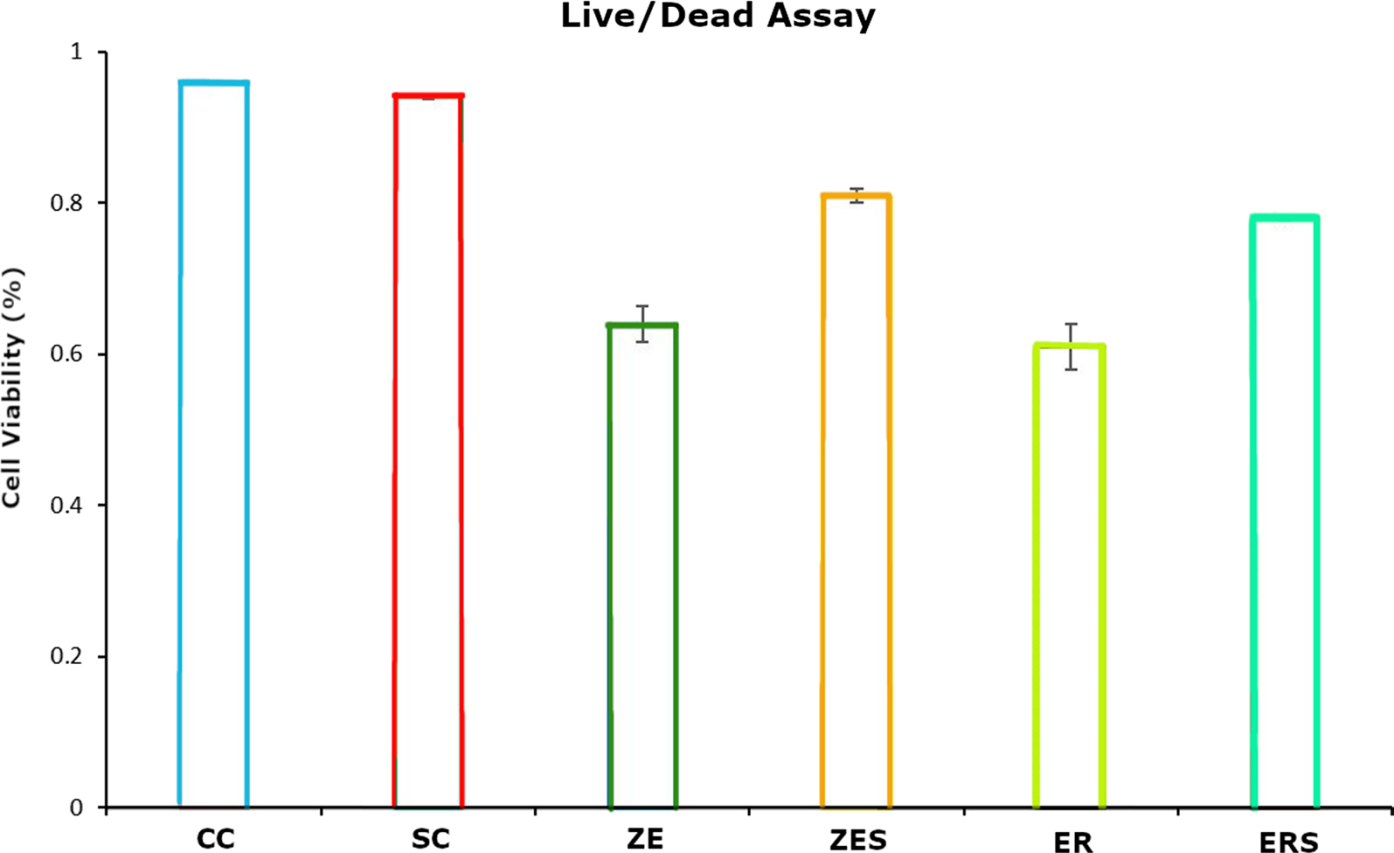
Data represent the mean ± standard deviation of experiments with triplicates (*p* < 0.05). Control groups (CC & SC) show the highest viable cells (%) while ZE and ER (without simvastatin) show the lowest percentage of viable cells

**Fig. 5:**
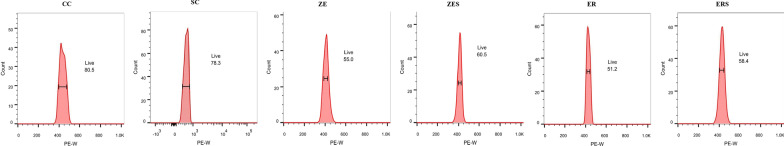
Cell viability by flowcytometry analysis. Histograms of flow cytometry shows the fluorescence intensity of calcian-AM. They represent the amount of cell proliferation and cell viability following 7 days. The histograms peaks indicates the percentage of live cells present in control and experimental groups

**Fig. 6:**
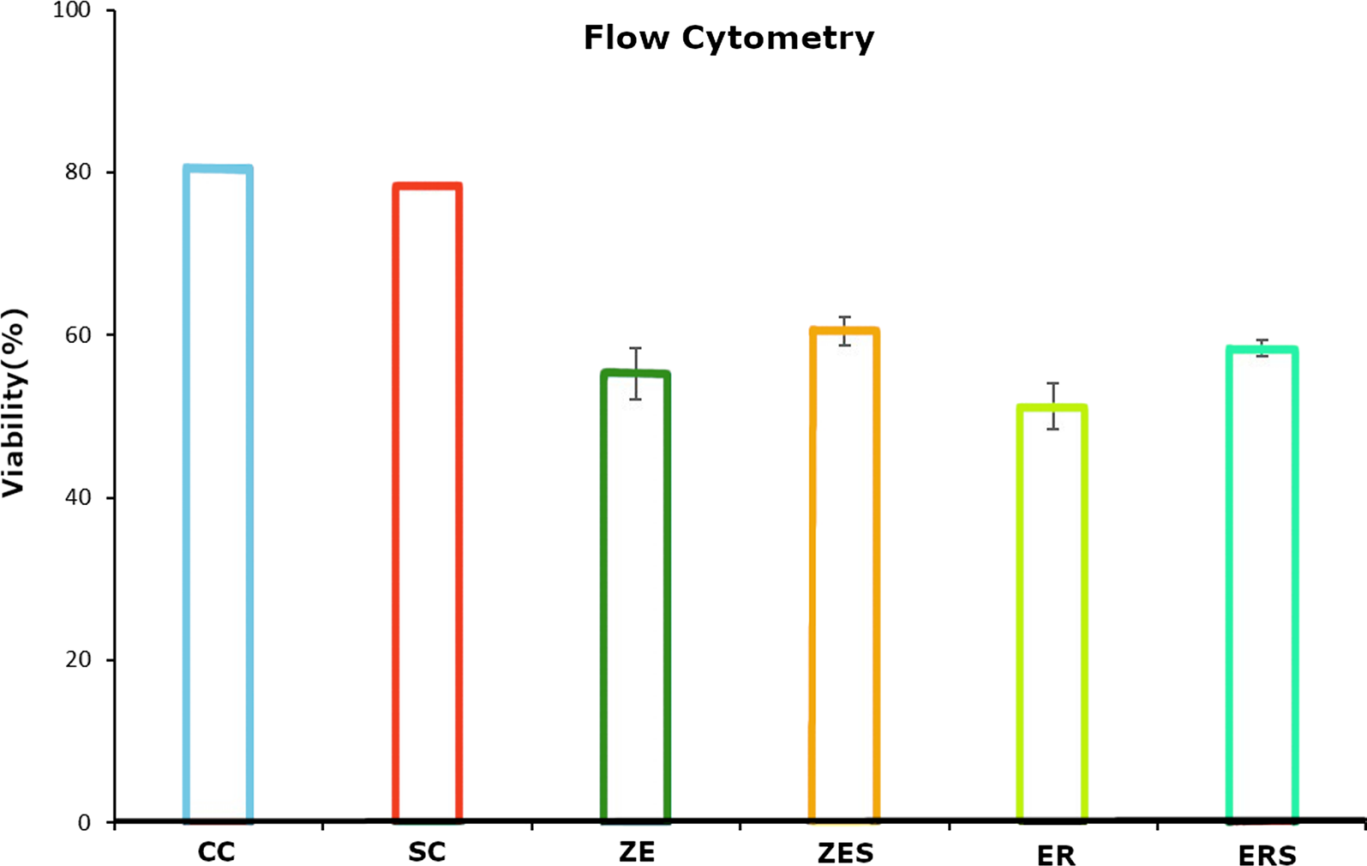
Data represents graphical representation of viability of cells among all the groups, following flow cytometer analysis.Control groups (CC&SC) show the highest while sealer groups without simvastatin (ZE&ER) show the lowest

The mean cell viability levels (%) and the mean cytokine (IL-6) expression [picogram/mL; (pg/mL)] for all the groups at different time periods of 0 h, 24 h and 7th day are given in Table 1 and 2. Graphical representation of the same are given in Figs. 1 and 2 respectively. Figures 3 and 4 represents live/dead assay of all the groups at different time periods (24 h and 7th day). Figures 5 and 6 represents histogram of flow cytometry analysis of all the groups.

**Table 2:** Mean ± SD of percentage of viable cells of all the groups at different time periods

Groups	0 h	24 h	7th day
CC	100.00 ± 0.000	100.00 ± 0.000	100.00 ± 0.000
SC	99.024 ± 0.001	92.740 ± 0.049	87.943 ± 0.017
ZE	96.042 ± 0.002^†^	73.315 ± 0.299	54.395 ± 0.354^†^
ER	99.242 ± 0.004	68.569 ± 0.516^†^	57.847 ± 0.021
ZES	99.513 ± 0.021	82.402 ± 0.371	75.933 ± 0.049
ERS	99.966 ± 0.014	81.380 ± 0.314	79.905 ± 0.048

n = 3, (*P* > 0.05); † Statistically significant. († represent the intergroup comparative values that are statistically significant against the control groups)

h-hours CC-Cell control; SC-Solvent control; ZE-Zinc Oxide Eugenol sealer; ER-EndoREZ sealer; ZES & ERS- Simvastatin incorporated Zinc oxide eugenol and EndoREZ respectively

### MTT assay

The cell control (CC) and solvent control (SC) groups were found to have increased cell viability at 24 h and 7th day.

ZE and ER showed decreased cell viability as compared to the control groups at 24 h till the 7th day. The cell viability for ZE and ER was 73.31% and 68.56% at 24 h which decreased to 54.39% and 57.84% after 7th day respectively. ZE was found to have least cell viability count (high cytotoxicity) by the end of the experimentation on 7th day. ZES and ERS were found to have higher cell viability counts than Z and ER at all the time periods. Cell viability for ZES and ERS were found to be 75.93% and 79.90% respectively by the end of the analysis. On 7th day, the level of cell viability can be stated in the descending order as-

CC > SC > ERS > ZES > ER > ZE.

### Live/dead staining and flow cytometry

In all the experimental groups, live cells were abundant (stained *green),* which increased from 24 h to day 7. Few dead cells (stained *red*) were observed in ZE, ER and least in ERS. The histogram peaks in flow cytometry denoted higher number of live cells in simvastatin incorporated sealers (ZES &ERS) compared to the sealer alone groups (ZE&ER).

### Cytokine detection

The cell control (CC) and solvent control (SC) groups for DMSO was found to have the least expression of IL-6 at all time periods.

ZE and ER showed significantly increased amount of IL-6 as compared to the control groups at 24 h till the 7th day. The amount of IL-6 for ZE and ER was found to be 25.49 pg/mL and 23.14 pg/mL respectively on the 7th day of evaluation, compared to 12.70 pg/mL and 14.68 pg/mL for ZES and ERS respectively. Therefore, lesser inflammation can be interpreted for simvastatin incorporated sealers compared to ZE and ER. On 7th day, the level of inflammation (detection of IL-6) can be arranged in the decreasing order as:

ZE > ER > ERS > ZES > SC > CC.

Simvastatin modified sealers (ZES & ERS) were found to have higher cell viability and lower IL-6 expression compared to ZE & ER.

## Discussion

Among the sealers tested in the present study, the addition of simvastatin significantly reduced the severity of sealer-induced cytotoxicity, hence, the null hypothesis was rejected. Cytotoxicity evaluation is inevitable with regard to biocompatibility of a newly modified material that is being considered for clinical usage, though the additive component is biocompatible on its own [28]. The literature evidence shows that a considerable variation exists in the outcomes of studies testing the cytotoxicity of the root canal sealers and the effects of simvastatin. This could be attributed to the assessment at different dilutions and concentrations, different techniques (cell cultures systems or animals), different composition of incorporated materials and evaluation at different time intervals. Root canal sealers are inserted into the canal in a freshly mixed state. During the unset and unpolymerized condition, the unreacted or partially reacted components may leach out provoking a local response. And, the leaching of potentially toxic components may continue even after setting of the material. Hence different time intervals were studied to evaluate whether these materials remain cytotoxic and induce inflammation or whether they lose the potential and heal [28, 29]. In order to analyse the cytotoxicity levels solely by the experimental materials and to remove a confounding factor, two separate control groups were added for comparison namely cell control (CC) and the solvent control (SC) for DMSO, since it has been found to show some amount of cytotoxicity on its own [30–32].

The observations of the present study signify the high level of cytotoxicity and increased expression of IL-6 of both ZE and ER in freshly mixed state (Tables 2 and 3; Figs. 3 and 5). This is in accordance with previous studies that observed that zinc oxide eugenol and Endo REZ presented significant cytotoxicity to the cultured cells [33, 34]. ZOE sealers being highly water soluble, dissolution of the material occurs invariably when it comes in contact with the tissue fluids (a reaction seen commonly with materials that set by an acid base reaction, e.g. Tubli-seal).The release of unreacted components like zinc ions, benzyl alcohol, methyl salicylic acid, and rosin could have contributed to cytotoxicity and inflammation at 24 h. Additionally, the release of free eugenol from the freshly mixed paste could have interfered with the cytoplasmic membrane, inhibiting cell respiration, contributing to cytotoxicity and increased IL-6 release in ZE [6].

**Table 3:** Mean ± SD of cytokine (IL-6) expression (pg/mL) of all the groups at different time periods

Groups	0 h	24 h	7th day
CC	0.1693 ± 0.139	0.1119 ± 0.095	0.5476 ± 0.010
SC	0.2643 ± 0.186	0.8024 ± 0.634	0.9714 ± 0.557
ZE	0.8500 ± 0.603	18.7000 ± 0.622^†^	25.4952 ± 0.307^†^
ER	0.9071 ± 0.545	18.6809 ± 0.267^†^	23.1476 ± 0.646
ZES	0.6167 ± 0.455	10.5190 ± 0.657	12.7048 ± 0.578
ERS	0.2833 ± 0.255	9.5571 ± 0.495	14.6810 ± 0.825

n = 3, (*P* > 0.05). ^†^Statistically significant. (^†^Represent the intergroup comparative values that are statistically significant against the control groups)

IL-6-Interleukin-6; pg/mL-picogram/millilitre

h-hours; CC-Cell control; SC-Solvent control; ZE-Zinc Oxide Eugenol sealer; ER-EndoREZ sealer; ZES & ERS- Simvastatin incorporated Zinc oxide eugenol and EndoREZ respectively

In ER, increased cytotoxicity in the first 24 h, could be due to initial outburst of elutes, namely the urethane dimethacrylate (UDMA), zinc and barium. UDMA, a known toxic agent is reported to cause intracellular glutathione depletion at low concentrations [6]. At high concentrations, it causes oxidative stress in the periapical region due to production of ROS [35]. Zinc and barium could have contributed by provoking a granulomatous reaction in the surrounding tissues [36, 37]. Up-regulation of cytokine, namely IL-6 was observed denoting persistent inflammation relating to the slow breakdown and extended setting time of EndoREZ sealer [38, 39]. This was evident with the lesser number of viable cells observed under fluorescent microscope and flow cytometry analysis for both the groups (Figs. 3, 4, 5, 6).

In actuality, time of exposure significantly influences the biocompatibility of dental resins. Though the cytotoxicity gradually lessened after 24 h, it persisted up to day 7. This could be due to continued release of materials over this period substantiating the further decrease in the percentage of cell viability noted in ER. Moreover, Ashraf et al, have stated that the cytotoxic properties of EndoREZ are less affected by its setting and that the material continued releasing highly toxic agents even after complete setting [40]. This explains the continued cytotoxicity exerted by ER in this present study.

At 24 h, among ER and ZE, decreased cell viability was observed in ER compared to ZE, though not statistically significant *(P* > *0.05).* Similar to our findings, Sousa et al., and Konjhodzic-Pric et al., reported high toxicity of ER [41, 42]. But at day 7, though both ER and ZE were cytotoxic, lesser cell viability was observed in ZE as compared to ER which was statistically insignificant (*P* > 0.05). Several histopathological and X-ray microanalyses of tissues in contact with ZOE-based materials, have revealed that they were more resistant to fragmentation for phagocytosis by interfering with the macrophage adherence, whereas ER requires more energy to be phagocytosed once extruded [43].

Simvastatin incorporated sealers, namely ZES and ERS showed increased cell viability at 24 h and 7th day compared to Z and ER (Table 2; Figs. 3, 4, 5, 6). This observation has been substantiated by the study done by Zhang et al., which reports improved biocompatibility of collagen coated polyethylene terephthalate scaffolds incorporated with simvastatin [44]. Simvastatin induced proliferation of bone marrow stromal cells, high alkaline phosphatase activity, osteoblastic differentiation, more mineralization deposition, and increased expression of osteoblast-related genes like osteocalcin, runt-related transcription factor 2, bone morphogenetic protein-2, and vascular endothelial growth factor than the other tested groups [44]. In addition Stein et al., and Varalakshmi et al., affirmed the positive effect of simvastatin on cellular differentiation, proliferation, and expression of growth factors, promoting various biological functions like angiogenesis and osteogenesis [44–48].

Sealer induced cytotoxicity results in the formation of reactive oxygen species (ROS), which in turn creates an oxidative stress in the tissues leading to an inflammatory response. Thus, cytotoxicity results in a cascade of inflammatory reactions in the tissues [6]. Mevalonate is the precursor of compounds that serve as lipid attachments to GTPases such as Rho, Rac, and Ras that mediate a number of inflammatory reactions such as NF-κB, activation of ROS and suppression of endothelial nitric oxide synthase which are dependent on isoprenoid production. It was observed that statins exert their effects by suppressing the downstream synthesis of molecules in the mevalonate pathways, mediated through the inhibition of small GTPase prenylation and isoprenoid production [15, 49]. This inhibition of isoprenoids supresses IL-6, IL-1ß, TNF-α (tumour necrosis factor) production [12, 49]. In this study, substantial reduction in the expression of IL-6 levels in ZES and ERS compared to ZE and ER explains the role played by simvastatin in reducing inflammation [15, 50–53]. These beneficial effects of simvastatin are concentration dependent [54]. 0.5 mg of simvastatin has been reported to fall within safe limits with potential to reduce inflammation and to induce new bone formation at resorption sites [15, 55–57].

Moreover, it is interesting to note that improved healing outcomes of endodontically treated teeth with preoperative lesions were noted in patients who were already under medication with simvastatin for their systemic condition. Statins are said to act by stimulating growth factors like VEGF, and subsequent inhibition of RANKL-induced NF-κB activation pathway. This in turn would suppress osteoclastogenesis and MMP-9 resulting in better healing potential, which would otherwise induce bone resorption [29, 58]. Hence the local delivery of simvastatin as designed in this study would by all means have a better effect on the tissues. Despite the favourable outcomes in the materials tested, further investigations are required to acquire valuable information on the physicochemical behaviour of this modified sealer formulation in order to be deemed appropriate for clinical use.

## Conclusions

Within the limitations of this in vitro study, it can be concluded that, the addition of simvastatin reduced the cytotoxicity and degree of inflammation of the sealers in freshly mixed state.

## Data Availability

The datasets used and /or analysed during the current study are available from the corresponding author.
